# Automated and Rapid Easy-to-Use Magnetic Solid-Phase Extraction System for Five Heavy Metals in Cereals and Feeds

**DOI:** 10.3390/foods11243944

**Published:** 2022-12-07

**Authors:** Wei Tian, Yonglin Liu, Songxue Wang, Jin Ye, Hongmei Liu, Yue Wang, Minghui Zhou

**Affiliations:** 1 Academy of National Food and Strategic Reserves Administration, Beijing 100037, China; 2 School of Environmental and Municipal Engineering, Qingdao University of Technology, Qingdao 266525, China

**Keywords:** easy-to-use MSPE system, carboxyl-functionalized magnetic beads, heavy metals, cereals and feeds

## Abstract

A rapid, accurate, and ecofriendly pretreatment plays an extremely important role prior to ICP-MS for heavy metal analysis. In order to improve the pretreatment efficiency, a high-throughput and automatic magnetic solid-phase extraction of five heavy metals (Cd, Pb, Mn, Cu, and Zn) was carried out by a magnet-controlled pretreatment system with an ecofriendly diluted acid as an extracting agent and carboxyl-functionalized magnetic beads as a pretreatment material. Key conditions, including the pH, adsorption time, and eluent solution, were optimized. The time for purification and enrichment was only 8 min. The adsorption capacities of the carboxyl-functionalized magnetic beads were in the range of 152~426 mg g^−1^. The preconcentration factor of Cu was 40, and others were 200. In the optimal conditions, the limits of detection for Mn, Zn, Cd, Cu, and Pb by ICP-MS were 3.84, 2.71, 0.16, 11.54, and 6.01 ng L^−1^, respectively. The percentage recoveries were in the range of 80~110%, and the relative standard deviations were less than 3%. The developed method was in good agreement with traditional standard microwave digestion. Additionally, the designed system could simultaneously process up to 24 samples within 22 min, reducing the time to less than 1 min/sample. Thus, the proposed auto-MSPE-ICP-MS method was successfully applied to analyze five heavy metals in cereals and feeds with a simple operation and high precision, safety, and reliability.

## 1. Introduction

Human uptake of most essential and/or non-essential heavy metals occurs mainly via the food chain. Cereals are the most abundant and basic food consumed by humans and animals [1]. However, the cultivated staple crops may be contaminated through the absorption of a variety of metals present in the air, water, or soil [2,3]. Some non-essential metals can show toxic properties, even at low concentrations. Cadmium (Cd) has a long half-life period and carcinogenic effects and is included in group I, according to the international agency for research on cancer [4]. Lead (Pb) has serious chronic toxicity to organisms and is defined as probably carcinogenic to humans [5,6]. Some potential toxic heavy metals such as manganese (Mn), copper (Cu), and zinc (Zn) are necessary nutrients for humans and animals in appropriate quantities. Mn is essential for the proper functioning of several enzymes. It takes part in protein synthesis, digestion, and energy production [7,8,9]. Zn is one of the most basic heavy metals in the immune system. It helps in maintaining normal DNA and protein synthesis [10]. Cu is required for the functioning of enzymes that are responsible for the renewal of cells, tissues, and bones [11]. Despite all these benefits, the intake of these elementals should not exceed their permissible limits as specified by regulatory agencies because when their intake is more than needed, they accumulate in various organs and cause various disorders [12]. Consequently, monitoring the concentrations of toxic and potential toxic heavy metals in cereals and their related products is of great importance from the toxicological and nutritional point of view [13].

To simultaneously quantify multiple heavy metals usually requires a highly sensitive multielemental technique, such as inductively coupled plasma mass spectrometry (ICP-MS) [14]. However, the accuracy of ICP-MS largely depends on the pretreatment of the sample. The pretreatment, as a leading role, can help to change target metals from solid to liquid, eliminate impurities, and improve sensitivity [15]. Usually, the classic method is digestion with a concentrated acid, which is time-consuming and dangerous for operators. The magnetic solid-phase extraction (MSPE) technique has several advantages compared with the classical method, namely saving time, a high enrichment factor, rapid phase separation, and a low consumption of acid solvents [16,17]. Up to now, some researchers have focused on MSPE based on different materials [18,19], but they still need manual operation and manual transfer. Few attempts have been made for automation in order to eliminate the manual procedures.

The motivation for this study was to develop an automatic MSPE method for the simultaneous clean-up and detection of five key heavy metals in cereals and feeds to improve the analytical potential of ICP-MS, that is, the development of a multifunctional platform that would combine the extraction, adsorption, washing, and elution processes into a single step. In our previous research [20,21], diluted acid was proven to have a relatively high extraction rate for some common metals. Therefore, in this platform, diluted acid and functional magnetic beads (MBs) were used in the design of a multielement automatic procedure. Carboxyl group MBs were selected as a sorbent material for the high-affinity functional groups on the surfaces with immense surface areas [22]. The MBs recognize and enrich the metals apart from unbound impurities, and dilute acid can destroy the bond, releasing the metals back to the solution for convenient subsequent detection [23]. To the best of our knowledge, this is the first report combining the advantages of a multifunctional automated MSPE pretreatment method with the high sensitivity and accuracy of ICP-MS for the determination of Cd, Pb, Mn, Zn, and Cu in cereals and feeds. The developed method proves to be convenient and accurate for the routine detection of Cd, Pb, Mn, Zn, and Cu in cereals and feeds.

## 2. Experimental Section

### 2.1. Reagents and Samples

All reagents were guaranteed-grade. Nitric acid, sodium hydroxide, cyclohexane, span-85, sodium carbonate, and ethanol were purchased from the Beijing Chemical Reagent Research Institute (Beijing, China). Toluene, dimethylformamide (DMF), succinic anhydride, and 3-aminopropyl trethoxy silane (APTES) were supplied from Shanghai McLean Biochemical Technology Co., Ltd. (Shanghai, China). The Fe_3_O_4_@SiO_2_ was purchased from Suzhou beaver Co., Ltd. (Jiangsu, China). Argon (99.9999%) was purchased from Beiwen gas manufactory (Beijing, China). Cd^2+^, Pb^2+^, Mn^2+^, Cu^2+^, and Zn^2+^ standard solutions were purchased from the National Institute of Metrology (Beijing, China). Animal feeds (sheep forage, cattle feed, chicken feed, and DDGS) were purchased from online shops. Cereals (rice, wheat, corn, and beans) were prepared by the Academy of the National Food and Strategic Reserves Administration (Beijing, China). 

### 2.2. Apparatus and Materials

The samples were ground by a grinder (Fritsch, Idar-Oberstein, Germany). An FE28 model pH meter (Mettler Toledo, Zurich, Switzerland) was used for the pH measurements. A multitube vortex mixer (LPD2500, Leopard, Beijing, China) was used to mix and extract. The automated magnet-controlled pretreatment instrument was homemade. Cd, Pb, Mn, Zn, and Cu were quantified by ICP-MS (8900CX, Agilent, Santa Clara, CA, USA). A Fourier-transform infrared spectroscope (FTIR, Thermo Fisher Scientific Nicolet IS5, Waltham, American), a scanning electron microscope (SEM, model LEO 440, Cambridge, United Kingdom) equipped with an energy-dispersive spectrometer (EDS, model Oxford link ISIS 300, Cambridge, United Kingdom), and an XRD apparatus (Bruker D8 Advance, Karlsruhe, German) were used for the characterization of the MB composite. A microwave digestion instrument (TOPEX, PreeKem, Shanghai, China) equipped with Teflon-PFA vessels was used for sample digestion.

### 2.3. Synthesis and Characterization of Functional-Group-Modified MBs

The carboxyl-functionalized MBs were synthesized according to the two-step sequence synthesis method. Briefly, 10 mg of Fe_3_O_4_@SiO_2_ was dispersed into the mixed solution of 12 mL of DMF and 8 mL of toluene (anhydrous). Then, 1 mL of APTES was added slowly and stirred mechanically for 24 h at room temperature while protected by nitrogen. After that, the solution was cleaned with toluene three times with the assistance of a magnet to obtain amino magnetic beads, which were stored in 10 mL of toluene for later use. Next, 40 mg of succinic anhydride was added to the above solution, protected by nitrogen, and magnetically stirred at 80 °C for 12 h. The precipitate was cleaned with toluene and absolute ethanol three times to remove any unreacted chemicals and dried in a vacuum at 55 °C for 12 h to obtain carboxyl magnetic beads. The synthesis of modified MBs was analyzed by FTIR, SEM, and EDS to characterize the physical and chemical properties. 

### 2.4. Automated Magnet-Controlled Pretreatment System

**Figure 1:**
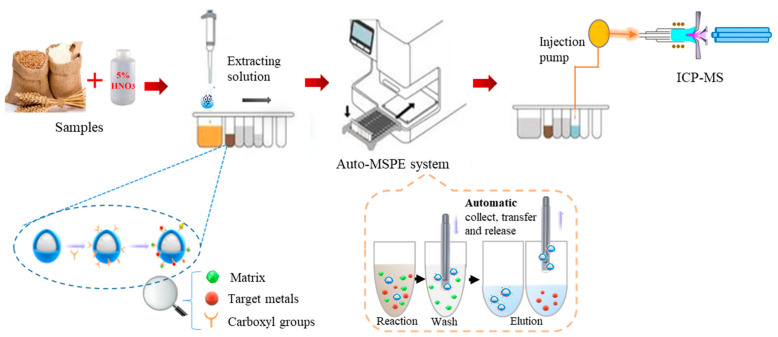
The process of the automated magnet-controlled pretreatment system.

The design of the automatic processes mainly included the magnetic bead transfer, adsorption, elution, washing, and recycling. The realization of these functions primarily depended on the magnet and the magnetic sticks inside the instrument. Subsidiary kits provided all the necessary reagents containing the sample diluents, elution buffer, and washing buffer. The process of the automated magnet-controlled pretreatment system is displayed in Figure 1. By analyzing the controlled pretreatment conditions and adjusting the subsidiary kits’ components (Table S1), the most optimal execution of the automated system could be ensured. 

An automated magnet-controlled pretreatment of five heavy metals from samples was carried out based on the following steps in batch mode: First, 1 mL of a sample extraction or standard solution supernatant was added to the first well, which had been pretreated with NaOH to adjust the pH to 6.0. Then, the 50 μL MBs in the second well were transferred into the first well to capture heavy metals for about 8 min. After that, loaded MBs were collected and diverted to the third and fourth wells to clean up the impurities on the surface with water. The fifth well was prefilled with diluted acid, which was used to elute the heavy metals absorbed by the MBs. The purification and concentration of pretreatment were completed in this step. After all the above procedures were completed, the unloaded MBs were returned to the second well for recycling and reuse (Figure S1). Finally, the sample treatment solution was used for detection.

### 2.5. Optimization of the Experimental Variables

To determine the optimal conditions for the best performance of the automated magnet-controlled pretreatment system and the modified MBs, several parameters were assessed in triplicate using a standard solution containing a 50 ng mL^−1^ concentration of five heavy metals. For the initial adsorption pH, the first well was investigated over the range of 2~10 by controlling the usage of NaOH. For the adsorption time, it was inspected from 0.5 to 60 min. Additionally, the influences of different eluents were examined on the recoveries of analytes. The eluent solutions were prepared from HCl, HNO_3_, EDTA, NaOH, and water at different concentrations.

The values of the adsorption capacity were calculated with the following procedure: Exactly 0.1 g of MBs were taken into the second well, and 500 ng mL^−1^ of examined metals were added into the first well with 1 mL. After the bath technique for pretreatment, the elute solution was analyzed by ICP-MS. The recovered concentration represented the adsorption capacity. In order to investigate the maximum preconcentration factor (PF), the developed extraction steps were applied to the working solutions. Exactly 10 mL working solutions containing 50 ng mL^−1^ multi-heavy-metal solutions were eluted by 2, 1, 0.5, 0.25, 0.1, and 0.05 mL of 5% HNO_3_.

### 2.6. Application to Cereals and Their Products

All the samples were grounded before analyzing. A portion (1.00 g) was weighed into a 10 mL tube. Then, 5 mL of 5% (*v*:*v*) HNO_3_ was added and shaken for 8 min. After natural deposition for 1 min, supernatants were collected and added to the automated pretreatment for purification and then quantified by ICP-MS.

Inductively coupled plasma mass spectrometry (ICP-MS) with an electrospray ionization source was operated in no-gas mode. The optimal parameters were set at an RF power of 2300 W, a torch-H of 0.3 mm, a torch-V of 0.4 mm, a carrier gas flow rate of 0.76 L min^−1^, a makeup gas flow rate of 0.45 L min^−1^, a sampling depth of 8.0 mm, a nebulizer pump flow rate of 0.10 rps, an integration time of 0.3 s point^−1^, and target masses of ^111^Cd, ^208^Pb, ^55^Mn, ^63^Cu, and ^66^Zn. The limit of detection (LOD), limit of quantitation (LOQ), recovery, specification, and repeatability were evaluated by the proposed analytical platform. 

## 3. Results and Discussion

### 3.1. Physical and Chemical Properties of Materials

**Figure 2:**
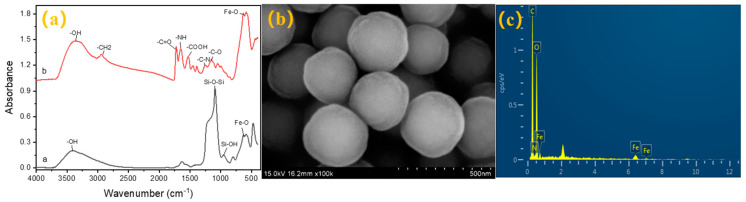
(**a**) FTIR spectra of Fe_3_O_4_@SiO_2_ and Fe_3_O_4_@COOH; (**b**) SEM photo of Fe_3_O_4_@COOH; (**c**) EDS of Fe_3_O_4_@COOH.

The physical and chemical properties of the prepared composites were characterized by FTIR, SEM, and EDS. As Figure 2a shows, the stretching vibration peaks, including the -OH (3408 cm^−1^) bond, Si-OH (953 cm^−1^), and the Fe-O (588 cm^−1^) bond, appeared in the basic composite, and the final FTIR spectrum had the same peaks as the raw material. In addition, the peaks of the -N-H (1651 cm^−1^), -C=O (1723 cm^−1^), and -COOH (1531 cm^−1^) belonging to carboxyl were found in the final composite as well. These data indicated that the synthesis was successful and that the carboxyl group was successfully coupled to the magnetic beads [24]. 

The SEM gave us information about the morphological structure and size. The result (Figure 2b) showed that the composite presented a round core–shell structure and a uniform size. The diameters averaged 15 μm. Moreover, the EDS was employed to evaluate the components of the material. The spectrum (Figure 2c) showed that the magnetic nanoparticles were mainly composed of Fe, C, and O, which roughly verified the existence of Fe_3_O_4_. All the above characteristics indicated that the material was synthesized successfully [25]. 

### 3.2. Effect of Initial pH

**Figure 3:**
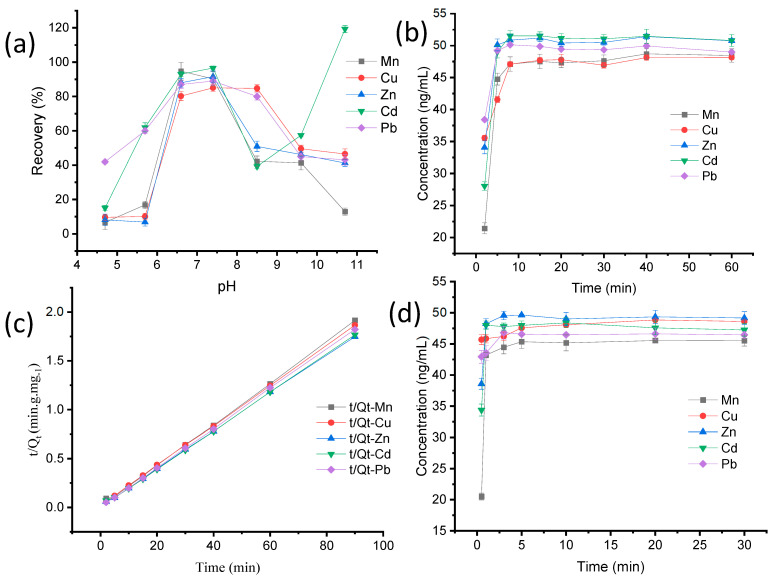
(**a**) Effect of initial solution pH; (**b**) Effect of adsorption time; (**c**) Pseudo-second-order kinetic model for adsorption; (**d**) Effect of the eluent time for Mn, Cu, Zn, Cd, and Pb.

The chemisorption of the coordination bond interaction has a dominant effect on adsorption [26]. Therefore, the acidity of the solution can change the MB surface charge, which affects the binding mechanism of heavy metals onto the solid phase. The concentration of all metals was very low at pH values from 2 to 5, while from 6 to 8 it was above 80%, and it dropped above 9. This phenomenon was consistent with previous reports. The adsorption mainly took place on the complexation with COOH, and H may be protonated at a low pH, while the protonated H may have electrostatic repulsion with the target metal cations [27]. Considering the results (Figure 3a), a pH of 7 was chosen for the solution in the experiments.

### 3.3. Effect of Adsorption Time 

The adsorption time affects the adhesion mechanism of ions [28] The effect of adsorption time was investigated from 0.5 to 60 min. The experiments were conducted three times with a 50 ng mL^−1^ mixed solution of five heavy metals. Referring to Figure 3b, the adsorption amount increased rapidly in the first 5 min, and the equilibrium was reached after 8 min. To describe the kinetics of heavy metal adsorption on the material, pseudo-second-order models were fitted according to the equation

(1) $$q_{t}=\frac{{k\;q}_{e}^{2}\;t}{{1+k\;q}_{e}t}$$

where K (min g mg^−1^) is the rate constant of adsorption and q_t_ (mg g^−1^) is the adsorption capacity at a given time, t. All the heavy metals followed the pseudo-second-order model (Figure 3c) because the regression correlations (R^2^) were approximately equal to 1.0, suggesting that the rate-limiting step was governed by surface attraction.

### 3.4. Influences of the Eluent Condition 

The elute time was also an important factor for evaluating the efficiency and the potential reusability. Therefore, after eluted for 0.5 to 20 min, and the concentrations are shown in Figure 3d. According to the results, the desorption amount increased rapidly in the first 3 min and thereafter tended to become parallel to the horizontal axis. 

**Figure 4:**
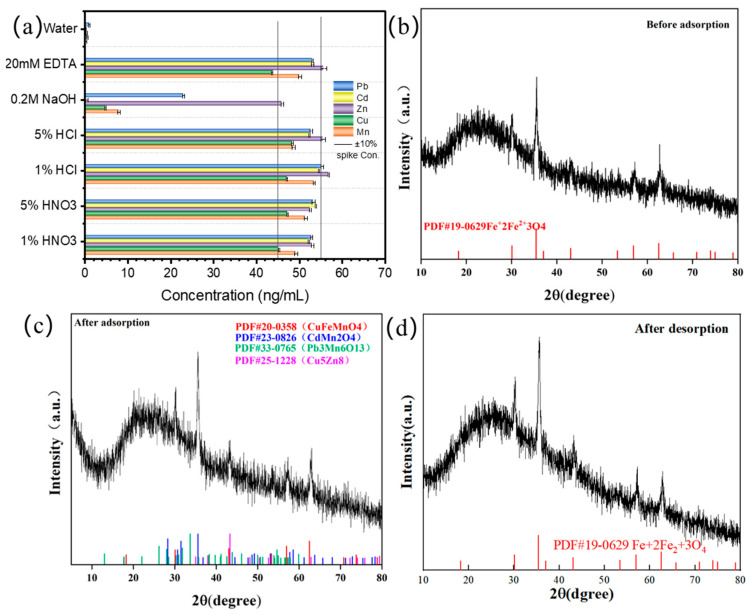
(**a**) Effect of the eluent condition for Mn, Cu, Zn, Cd, and Pb; (**b**) XRD pattern before adsorption; (**c**) XRD pattern after adsorption; (**d**) XRD pattern after desorption.

The influences of different eluents are shown in Figure 4a. For all the metals, the concentrations with 5% HNO_3_ fell in appropriate range and were more stable than the others. Therefore, in this study we preferred 5% HNO_3_ as the eluent. Meanwhile, the water could not desorb any heavy metals, so we had sufficient evidence to choose water as the washing solution to remove nonspecific adsorption in the 3rd wells of the kits. 

In order to observe the change in the material between adsorption and desorption from a microscopic perspective, the phases of the materials were identified by XRD, as shown in Figure 4b–d. Before adsorption, the main peaks were in good agreement with the standard characteristic peaks of the original Fe_3_O_4_, and there was no heterogeneous peak, which represented the high purity of the crystal structure. In that state, the diffraction angles corresponding to these peaks were 30.095°, 35.422°, 43.052°, 56.942°, and 62.515°, respectively. After adsorption, the main peaks were in good agreement with the standard characteristic peaks of CuFeMnO_4_ (30.063°, 43.037°, and 62.491°), CdMn_2_O_4_ (35.597° and 57.739°), Pb_3_Mn_6_O_13_ (30.272° and 57.244°), and Cu_5_Zn_8_ (35.05° and 43.297°). After desorption for 3 min, the material returned to the same characteristics as the original Fe_3_O_4_. In comparison to previous work [29], all these collected data showed that the adsorption and elution processes were occurring completely and that the MBs had a great potential for reusability from a microstructure perspective.

### 3.5. Clean-Up Performance

**Figure 5:**
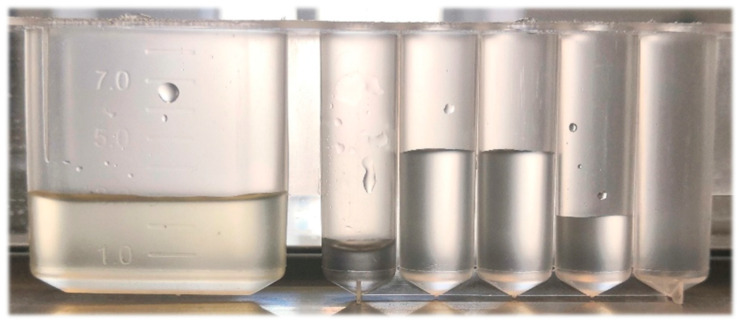
The states in each well of the kit after the clean-up procedure with MBs. From left to right: sample well (yellow and muddy), MB well, two washing wells, and elution well (limpid).

The auto-MSPE for the clean-up of complexed grain and feed samples had the potential to be a simple and time-saving operation because it completed the purification of the pretreatment without any manual operation, such as digestion, heating, acid driving, or centrifugation. Meanwhile, it can effectively remove impurities that may block the ICP-MS pipeline. Figure 5 shows that after the pretreatment of auto-MSPE, the final well was much clearer and more translucent than the original sample solution. The ICP-MS analysis further confirmed this observation, as there were no errors during the normal running of the program. 

### 3.6. Method Validation

The calibration curves of the analytes were fitted, and the linear ranges were from 0.1 to 400 ng mL^−1^, with regression coefficients (R^2^) in the range of 0.9983~0.9998 (Figure S2). Five regression equations corresponding to the five metals were obtained, as shown in Table 1, and the equations showed strong linear correlations between the concentration and signal response. The preconcentration factors were calculated according to the recovery results. The preconcentration factor of Cu was 40, and the others were 200. Comparing to Molaei et al. [30], the proposed method had the same PF for Cu but a significant improvement for the others. In view of the detection limits, the ratios of three times the standard deviations of eleven blank samples were calculated as the LODs and the ratios of ten times the standard deviations of eleven blank samples were calculated as the LOQs with preconcentration factors. The LODs were found in the range of 0.16~11.54 ng L^−1^ as 3.84 ng L^−1^ for Mn, 2.71 ng L^−1^ for Zn, 0.16 ng L^−1^ for Cd, 11.54 ng L^−1^ for Cu, and 6.01 ng L^−1^ for Pb, which can satisfy the need for routine determination. The precision of this method was also evaluated by an analysis of the standard reference materials (GBWE 100377) on five batches with RSD < 3%, which confirmed the good repeatability of this method (Table S2). 

### 3.7. Application to Real Samples

The developed auto-MSPE procedure was introduced for the preconcentration and detection of five heavy metals from rice, wheat, corn, beans, sheep forage, cattle feed, chicken feed, and the feeds’ raw materials (DDGS). In order to prove the accuracy of the auto-method using MBs, the traditional microwave digestion method was used for comparison. Table 2 shows the data of average recoveries ± standard deviations. As can be seen, good agreement was obtained between this auto-method and the microwave digestion method. All recoveries for the five metals in eight kinds of real samples were above 80%, and no significant differences were observed (Student’s *t*-test and 95% confidence level). The closeness of the values between the two method shows the accuracy of this developed method.

### 3.8. Comparison with Other MSPE Methods

The performance of this method was compared with some reported techniques, as shown in Table 3. For the adsorption capacity, the values were obtained with the plateau concentration per gram of MBs. The adsorption capacities were found to be 152 mg g^−1^ for Mn, 426 mg g^−1^ for Cu, 277 mg g^−1^ for Zn, 229 mg g^−1^ for Cd, and 402 mg g^−1^ for Pb, which were better than those in previous reports. For the MSPE extraction time, this proposed method was at the same level as the present approaches, but for the whole pretreatment time this work showed significant advantages by saving about a quarter of the time. From extraction to detection, this proposed method can process up to 24 samples in a batch within only 22 min. Furthermore, this method showed excellent sensitivity. The LODs are better than or comparable to those of other works. In addition, the proposed method realized an automatic pretreatment with the pretreatment technique of diluted acid extraction, which had an easy operation, saved time, had no digestion, and was human-friendly. Therefore, our work could improve efficiency and save labor in the detection of heavy metals in cereals, feeds, and related products, which is of great significance to ensuring food safety. 

## 4. Conclusions

The developed method proves to be convenient and accurate for the routine detection of Cd, Pb, Mn, Zn, and Cu in cereals and feeds. In this work, a simultaneous automated pretreatment method of magnetic solid-phase extraction with Fe_3_O_4_@COOH coupled with ICP-MS was developed for analyzing five heavy metals in cereals and feeds. The optimized method showed that the MSPE eliminated complex pretreatment steps and greatly reduced both the time and the danger caused by manual operation. The Fe_3_O_4_@COOH had a good adsorption capacity and a fast adsorption ability compared with a traditional pretreatment. The method validation exhibited a satisfactory linearity, LOD, recovery, and repeatability. The automation and high throughput of this method make it an effective way for laboratories to obtain multielement data quickly and accurately from cereals, feeds, and other dairy products.

## Figures and Tables

**Table 1:** Analytical parameters of the proposed method.

Statistical Parameters	Cu	Zn	Mn	Cd	Pb
Linear ranges (μg L^−1^)	0~200	0~400	0~400	0~400	0~400
Regression equations	y = 0.930x − 2.07	y = 1.068x + 0.98	y = 0.955x + 0.54	y = 1.077x − 2.03	y = 1.066x − 2.18
Coefficients (R^2^)	0.9983	0.9995	0.9992	0.9998	0.9991
RSD (%)	1.5	1.7	2.1	2.5	1.2
LOD (ng L^−1^)	11.54	2.71	3.84	0.16	6.01
LOQ (ng L^−1^)	38.08	8.94	12.67	0.53	19.83

**Table 2:** The analyte recoveries in rice, wheat, corn, beans, feeds, and feeds’ raw materials (*n* = 3).

Sample	Mn	Cu	Zn	Cd	Pb
Rice	88.8 ± 2.1	88.7 ± 0.8	82.8 ± 2.5	91.7 ± 1.6	82.7 ± 2.4
	86.3 ± 2.4	83.6 ± 1.2	84.7 ± 1.4	97.8 ± 1.4	89.0 ± 1.6
	82.0 ± 3.0	89.8 ± 2.3	83.4 ± 1.3	92.7 ± 1.5	88.8 ± 1.7
	88.5 ± 1.9	86.0 ± 1.5	81.1 ± 2.1	89.8 ± 2.1	86.8 ± 2.5
	88.9 ± 1.0	89.0 ± 1.2	83.8 ± 2.0	90.1 ± 2.8	BDL
	88.0 ± 0.9	92.1 ± 2.5	81.2 ± 1.6	94.2 ± 3.2	98.9 ± 3.4
Wheat	88.3 ± 2.3	85.6 ± 3.4	89.8 ± 3.5	88.0 ± 3.5	91.8 ± 3.2
	85.0 ± 3.0	91.7 ± 3.2	87.7 ± 3.6	90.8 ± 3.7	95.8 ± 1.5
	95.3 ± 1.5	98.6 ± 1.8	87.1 ± 2.9	80.1 ± 2.9	85.2 ± 3.8
	88.3 ± 2.4	91.0 ± 2.7	81.8 ± 3.2	89.2 ± 2.5	91.2 ± 2.5
	92.4 ± 3.0	92.9 ± 1.9	88.2 ± 3.7	89.8 ± 1.8	93.8 ± 2.4
	82.8 ± 3.1	98.8 ± 1.5	85.7 ± 2.8	86.2 ± 1.5	91.1 ± 1.7
Corn	83.4 ± 2.6	91.5 ± 2.8	82.4 ± 3.1	87.6 ± 3.3	91.2 ± 1.1
	80.3 ± 3.8	91.8 ± 3.2	85.8 ± 3.4	94.6 ± 2.1	94.1 ± 1.4
	87.4 ± 1.5	89.3 ± 3.0	85.9 ± 2.6	90.7 ± 1.1	94.0 ± 2.2
Beans	84.2 ± 1.2	91.0 ± 1.6	87.7 ± 2.8	93.1 ± 3.3	88.1 ± 2.0
	80.8 ± 1.0	90.6 ± 2.4	94.0 ± 1.9	91.2 ± 3.5	BDL
DDGS	85.5 ± 2.4	87.5 ± 2.2	87.2 ± 1.2	96.6 ± 3.6	92.0 ± 2.3
Sheep forage	80.8 ± 1.8	88.7 ± 3.0	82.8 ± 0.8	91.7 ± 2.8	92.7 ± 1.5
Cattle feed	86.3 ± 2.2	83.6 ± 1.6	84.7 ± 3.5	97.8 ± 2.7	89.0 ± 3.3
Chicken feed	106.3 ± 1.2	92.6 ± 1.6	87.4 ± 3.5	82.5 ± 2.1	102.5 ± 2.6

BDL: Below detection limit.

**Table 3:** Comparison of the proposed method with reported methods.

Detection Technique	Pretreatment Technique	Working Mode	MSPE	Heavy Metal	Pretreatment Total Time (min)	LOD (ng L^−1^)	Real Samples	Capacity (mg g^−1^)	Ref
AAS	Digestion	Manual	Fe_3_O_4_@AT@ED	Cd, Cu, Pb	1209	1080~1510	Beans	-	[29]
ICP-MS	Digestion	Manual	mGO/SiO_2_@coPPy-Th	Cu, Pb, Zn, Cr, Cd	116.5	150, 650, 230, 360, 210	Tomatoes, apples, water	201, 230, 125, 98, 80	[30]
ICP-MS	Digestion	Manual	MGO/SiO_2_@PANI-PPy	Cr, Pb	106.3	4.808, 3.401	Rice	188.9, 213.3	[31]
ICP-MS	Digestion	Manual	Fe_3_O_4_@SiO_2_@PAR	Cr, Cd, Pb	109	11.9, 0.8, 4.1	Human fluids	62.9, 56.6, 43.3	[32]
ICP-AES	Digestion	Manual	Fe_3_O_4_/HAP/GQDs	Cu	120	580	Food	-	[33]
ICP-MS	Filter	Manual	Fe_3_O_4_@UiO-66–NH_2_	Cd, Pb	300	-	Water	714.3, 370	[34]
ICP-MS	Diluted acid extraction	Automatic	Fe_3_O_4_@COOH	Cu, Zn, Mn, Cd, Pb	22	11.54, 2.71, 3.84, 0.16, 6.10	Cereals, feeds	426, 277, 152, 229, 402	This work

## Data Availability

Data is contained within the article or supplementary material.

## Supplementary Materials

The following supporting information can be downloaded at: https://www.mdpi.com/article/10.3390/foods11243944/s1, Figure S1: Routing of the automatic pretreatment system based on MSPE; Figure S2: The standard curves of Mn, Cu, Zn, Cd and Pb; Table S1: The sequence time for each step of the automatic pretreatment system; Table S2: The precision of the proposed method.
