# Supplementary material for: Automated and Rapid Easy-to-Use Magnetic Solid-Phase Extraction System for Five Heavy Metals in Cereals and Feeds
Source: Foods. 2022 Dec 7;11(24):3944. doi: 10.3390/foods11243944 (PMC9778536; doi:10.3390/foods11243944)
Supplement: Supplementary file 1 [file foods-11-03944-s001.zip › foods-2052939-supplementary.pdf]

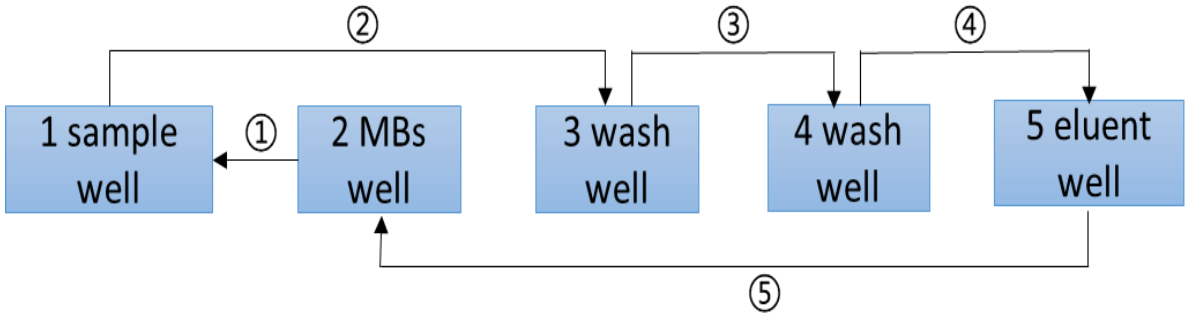

Figure S1. Routing of the automatic pretreatment system based on MSPE.

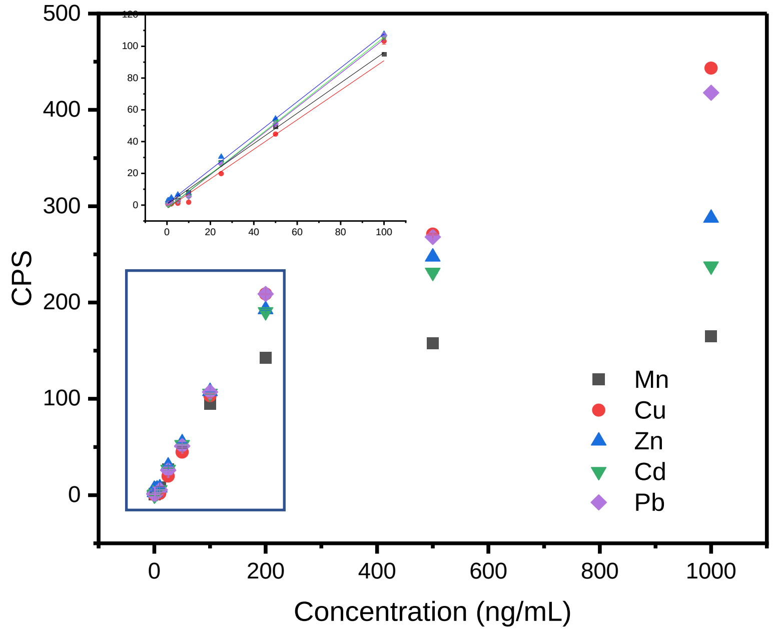

Figure S2. The standard curves of Mn, Cu, Zn, Cd and Pb.

Table S1. The sequence time for each step of the automatic pretreatment system.

Step	Sequence	Well	Mixing Time (min)	Transfer Time (min)	Mixing Frequency (Hz)
1	Absorption	1	8	2	7.5
2	Wash 1	3	1	1.5	6.5
3	Wash 2	4	1	1.5	6.5
4	Elution	5	3	0.5	7.5
5	Collection	2	0.5	0.5	6.5

Table S2. The precision of the proposed method.

Metal	Measurement Value (mg. kg^{-1}) (n = 5)					Average Value (mg. kg^{-1})	RSD (%)
Mn	2.411	2.581	2.510	2.438	2.536	2.495	2.81
Cu	2.947	2.871	2.852	2.881	2.932	2.897	1.41
Zn	1.943	2.039	2.074	1.945	2.011	2.002	2.89
Cd	0.230	0.223	0.218	0.219	0.213	0.221	2.88
Pb	0.208	0.197	0.195	0.202	0.197	0.199	2.91
